# Establishing the Association Between Osteoporosis and Peptic Ulcer Disease: A Systematic Review

**DOI:** 10.7759/cureus.27188

**Published:** 2022-07-23

**Authors:** Sneha Teresa Selvin, Sonu Thomas, Viktoriya Bikeyeva, Ahmed Abdullah, Aleksandra Radivojevic, Anas A Abu Jad, Anvesh Ravanavena, Chetna Ravindra, Emmanuelar O Igweonu-Nwakile, Safina Ali, Salomi Paul, Shreyas Yakkali, Prachi Balani

**Affiliations:** 1 Internal Medicine, California Institute of Behavioral Neurosciences & Psychology, Fairfield, USA; 2 Behavioral Neurosciences and Psychology, California Institute of Behavioral Neurosciences & Psychology, Fairfield, USA; 3 General Surgery, California Institute of Behavioral Neurosciences & Psychology, Fairfield, USA; 4 Internal Medicine, Saint Vincent Hospital, Worcester, USA

**Keywords:** bone fracture, bone mineral density, bone resorption, osteoporosis, proton pump inhibitor, helicobacter pylori, peptic ulcer disease

## Abstract

Osteoporosis is one of the most common metabolic bone diseases. Many studies were conducted to find the association between peptic ulcer disease (PUD), *Helicobacter pylori* infection, proton-pump inhibitor (PPI) use, and increased risk for fracture, but results remain ambiguous. We performed this systematic review to understand the association between PUD and osteoporosis. We comprehensively searched relevant articles on April 19, 2022, by exploring different databases including PubMed, PubMed Central (PMC), and Medline using relevant keywords. After applying inclusion and exclusion criteria and undergoing quality assessment, we retained 25 studies published in and after 2015. For our systematic review, we included a total of 5,600,636 participants. The studies included in our review demonstrated a significant association between PUD, *H. pylori* infection, and the risk of osteoporosis. Long-term PPI use was also found to be a risk factor for osteoporosis. Malabsorption of nutrients, increase in inflammatory cytokines, and alterations in hormone status were found to be the notable factors behind the association. Early management of *H. pylori* infection and cautious use of long-term PPIs may protect against osteoporosis. Further randomized controlled trials (RCTs) are necessary to establish a causal relationship.

## Introduction and background

Osteoporosis, also known as the “silent disease”, is one of the most common metabolic bone diseases characterized by reduced bone mineral density (BMD), impaired bone strength, increased bone fragility, and increased susceptibility to fractures, especially in hip and spine [1,2]. Osteoporosis has become a considerable health concern for individuals and societies [3]. The disability, morbidity, and mortality caused by osteoporosis impose a significant burden on affected individuals, their families, and the health care system [4]. Therefore, the potential burden of osteoporosis should be mitigated by identifying the risk factors and the population at risk of this disease [2].

Peptic ulcer disease (PUD) develops when the gastrointestinal protective mechanisms, such as bicarbonate and mucus secretion, are overwhelmed by the detrimental effects of gastric acid and pepsin [5]. The risk factors for PUD include *Helicobacter*​​​​* pylori* infection, nonsteroidal anti-inflammatory (NSAID) use, aspirin use, tobacco smoking, and a low level of physical activity [6]. PUD presents with ulcers along the stomach or duodenal lining, causing burning pain. It may result in complications such as ulcer perforation and internal hemorrhage [7].

Proton-pump inhibitor (PPI), one of the widely used drug classes in the world, is used for PUD management as well as for *H. pylori* eradication regimens [8,9]. PPIs are also one of the most commonly used off-label drugs, with 25%-70% of total prescriptions having no appropriate indication [10]. Despite excellent efficacy and trivial short-term side effects, rising concerns regarding long-term adverse effects of PPI use such as the risk of osteoporosis-related fracture, vitamin B12 deficiency, renal injury, *Clostridium difficile* infection, community-acquired pneumonia, and dementia is emerging [11]. Several studies have demonstrated a significant positive association between PUD, long-term PPI use, and increased incidence of osteoporotic fracture risk [2,12-19]. Therefore, this systematic review aims to explore the association between osteoporosis and PUD and understand the different mechanisms by which osteoporosis emerges as a complication of PUD.

## Review

Methods

We conducted this systematic review in accordance with the Preferred Reporting Items for Systematic reviews and Meta-Analyses (PRISMA) guidelines [20]. A comprehensive literature search of electronic databases, including PubMed, PubMed Central (PMC), and Medline, was conducted on April 19, 2022. We searched for relevant studies using generic keywords “osteoporosis” AND “peptic ulcer” and identified 227 studies. The relevant terms of Medical Subject Headings (MeSH) were used in combination, and we got 73,687 studies. We also reviewed the reference section of the retrieved articles for additional relevant studies that were possibly missed in the initial search and obtained 21 articles. After applying inclusion and exclusion criteria and screening the articles by title, abstract, and full text, 25 reports were available for quality assessment, after which all the studies were retained. Table 1 displays the search strategy results.

**Table 1:** Database Search Using Keywords and MeSH Search Strategy MeSH: Medical Subject Headings.

Keywords	Total articles	Inclusion/exclusion, duplicate removal	Screening by title, abstract, and full text	After quality check
Osteoporosis OR reduced bone mineral density OR increased bone resorption OR Cortical bone loss OR bone loss OR bone depletion OR trabecular thinning OR ( "Osteoporosis/pathology"[Majr] OR "Osteoporosis/physiopathology"[Majr] OR "Osteoporosis/prevention and control"[Majr] ) OR ( "Bone Resorption/pathology"[Majr] OR "Bone Resorption/physiopathology"[Majr] OR "Bone Resorption/prevention and control"[Majr] ) AND Peptic ulcer OR gastric ulcer OR duodenal ulcer OR gastric mucosal lining sore OR gastric sore OR "Peptic Ulcer/complications"[Majr] OR "Stomach Ulcer/complications"[Majr] OR "Duodenal Ulcer/complications"[Majr]	73,687	334	4	4
“osteoporosis” AND “peptic ulcer”	227	2	0	0

Inclusion Criteria

We included articles published in English between 2015 and 2022 involving human participants and articles in full-text format. Meta-analyses, systematic reviews, RCT, cohort studies, case-control studies, and traditional reviews were included.

Exclusion Criteria

Gray literature, case reports, case series, animal studies, overlapping studies, studies not written in English, studies with inaccessible full text, and studies published before 2015 were excluded.

Results

**Figure 1:**
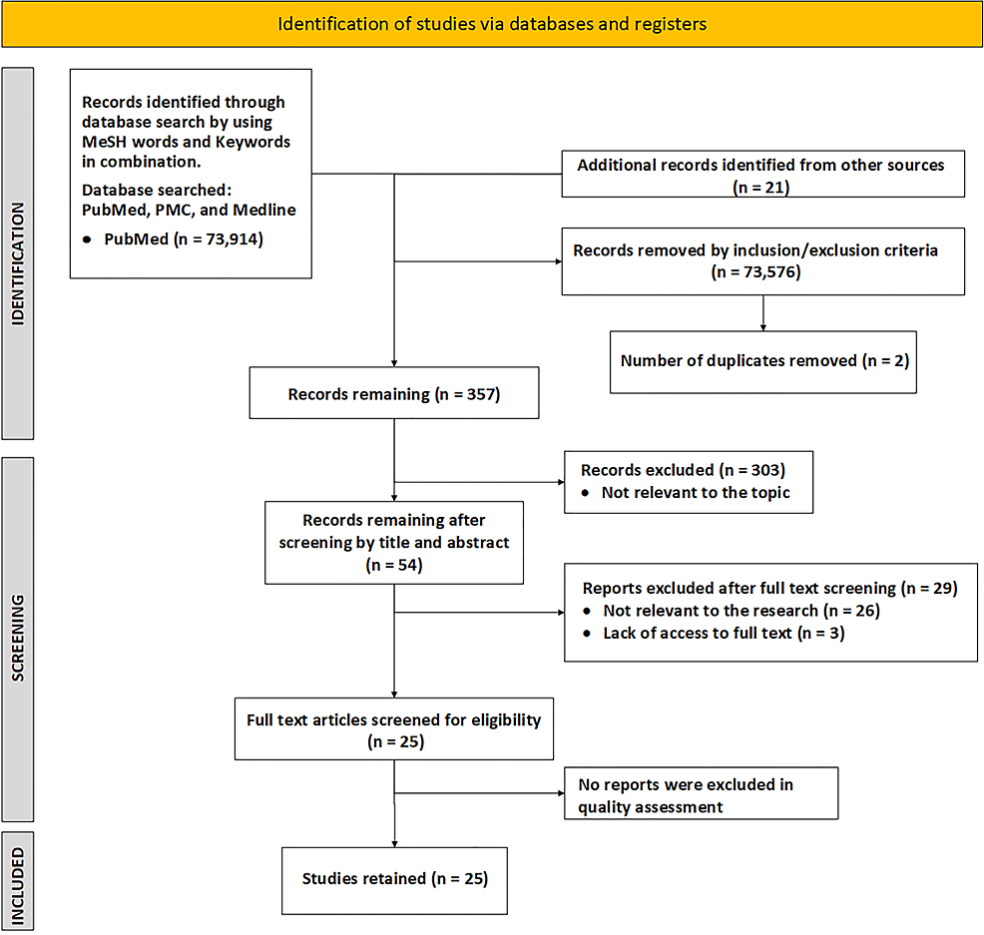
PRISMA Flowchart Showing the Study Selection PRISMA: Preferred Reporting Items for Systematic Reviews and Meta-Analyses; PMC: PubMed Central; MeSH: Medical Subject Headings.

We identified a total of 73,914 articles through search strategy and 21 articles from other sources identified mainly by reviewing the reference section of the retrieved articles. A total of 25 articles were kept after screening by title, abstract, and full text. After setting a 60% benchmark for quality assessment, all 25 studies were retained. We used the following quality assessment tools: Cochrane risk-of-bias tool (RoB 2) for RCT, Assessment of Multiple Systematic Reviews (AMSTAR) appraisal tool for systematic review and meta-analysis, Newcastle Ottawa Scale for observational studies (case-control and cohort studies), and Scale for the Assessment of Narrative Review Articles (SANRA) for traditional reviews. In our systematic review, a total of 5,600,636 participants were included. We identified one RCT, two meta-analyses, two systematic reviews and meta-analyses, 10 cohort studies, two case-control studies, and eight traditional reviews. Figure 1 shows the PRISMA flowchart.

Discussion

PUD is one of the most common upper gastrointestinal diseases and is found to have a significant association with the development of osteoporosis [2,7,12,15].

Peptic Ulcer Disease: The Pathophysiology

PUD is caused mainly by *H. pylori* infection, NSAID use, tobacco use, alcohol use, and low physical activity. PUD is characterized by gastric or duodenal ulcers along the mucosal lining [6,7]. *H. pylori* infection is a bacterial infection acquired during childhood via fecal-oral transmission, persists for life unless eradicated by treatment, and is responsible for 95% of duodenal ulcers and 85% of gastric ulcers [21]. In the stomach, *H. pylori* infection results in many pathophysiologic events including gastric acid neutralization, damage in the mucus layer, increase in proinflammatory interleukins (IL-1β, IL-6, IL-8, IL-17), tumor necrosis factor alpha (TNF-α), interferon gamma (IFN-γ), C-reactive protein (CRP), and anti-inflammatory (IL-4 and IL-10) cytokines. Also, *H. pylori* infection can lead to enhanced production of reactive oxygen species resulting in cell damage and changes in gastric structure and function, causing alterations in hormone production and secretion of acid and pepsin. In particular, *H. pylori* infection with virulent strains like CagA positive (positive for cytotoxin-associated gene A), which is associated with an enhanced inflammatory response, plays a critical role in the development of PUD [21,22].

PPIs, used for PUD management and *H. pylori* eradication, suppress gastric acid secretion by irreversibly inhibiting the gastric H^+^K^+^-ATPase pump on the parietal cells and have a very long duration of action with a gastric acid suppression effect that can last up to one week [8,23]. Hypochlorhydria/achlorhydria caused by PPI and *H. pylori* infection can result in chronic hypergastrinemia and malabsorption of nutrients like calcium (Ca), magnesium (Mg), iron (Fe), and vitamin B12 [23-25]. Thus, PUD caused by *H. pylori* infection is associated with a proinflammatory and hypochlorhydric/achlorhydric state which can result in malabsorption of nutrients and hormonal imbalance.

Osteoporosis: A Complication of PUD

Several studies have demonstrated a significant positive association between osteoporosis and PUD [2,12-19]. However, one study yielded a conflicting result regarding the association [26]. A cohort study by Wu et al. reported that the risk of osteoporosis was 1.85 times higher in PUD group when compared to non-PUD group (13.99 vs 5.80 per 1,000 person-years, respectively), with a significantly (p < 0.001) higher incidence and a faster development of osteoporosis in PUD group (3.6 years) when compared to non-PUD group (6.4 years) [2]. A similar association was reported by Choi et al. where the PUD group had a significantly (p < 0.001) higher rate of osteoporosis (27.3% [13,662/50,002]) than the control group (20.9% [10,440/50,002]) regardless of sex [12]. Also, the PUD group had an adjusted hazard ratio (HR) of 1.36 (95% confidence interval [CI] = 1.33 to 1.40) for osteoporosis [12]. Similarly, a cohort study done in Korea by Yoon et al. concluded that the risk of developing osteoporosis was greater in both men (HR = 1.72, 95% CI = 1.02 to 2.92) and women (HR = 1.62, 95% CI = 1.20 to 2.18) in the PUD cohort when compared to those in the control group [15]. A higher incidence rate per 1,000 person-years was reported in men (20.5%) and women (68.5%) who had PUD than in men (11.2%) and women (42.3%) with no PUD [15].

The relation between *H. pylori* infection and osteoporosis was discussed by Shih et al., who reported a higher risk of osteoporosis development in the early *H. pylori* treatment group (HR = 1.52, 95% CI = 1.23 to 1.89) and late *H. pylori* treatment group (HR = 1.69, 95% CI = 1.39 to 2.05) compared to the control group [13]. Also, both the early and late cohorts exhibited a higher risk of developing osteoporosis (HR = 1.69, 95% CI = 1.32 to 2.16 and HR = 1.72, 95% CI = 1.38 to 2.14, respectively) when followed up for less than five years. However, when the follow-up period was beyond five years, a higher incidence of osteoporosis was demonstrated only by the late eradication cohort (HR = 1.62, 95% CI = 1.06 to 2.47) owing to their chronic *H. pylori* exposure [13]. Thus, early eradication of *H. pylori* infection decreases the incidence of osteoporosis.

The medication used to treat *H. pylori* infection may be another cause of osteoporosis [13]. Based on available data, the US Food and Drug Administration (FDA) issued a drug safety warning in 2011 stating that patients who received PPI for a year or more and/or those who received high doses of prescription PPIs are at increased risk for hip, wrist, and spine fractures [27]. The association between PPI use and the occurrence of osteoporosis was revealed in a study by Wu et al., who concluded that there was a significantly higher risk of osteoporosis in PUD patients with PPI use (HR = 1.17, 95% CI  =  1.03 to 1.34) when compared to PUD patients without PPI use [2]. Compared to non-PUD patients without PPI use, a higher osteoporosis risk was found in non-PUD patients with PPI use (HR = 1.48, 95% CI  = 1.19 to 1.82), in PUD patients without PPI use (HR = 1.88, 95% CI = 1.75 to 2.01), and in PUD patients with PPI use (HR = 2.2, 95% CI = 1.91 to 2.52) [2]. This observation was replicated in an RCT by Jo et al., who identified that when elderly patients took PPI for more than eight weeks, their bone parameters were significantly altered due to osteoclast action enhanced by PPI use, exposing them to a higher risk of fracture [14]. A comparable observation was noted in a case-control study by Park et al. in which the cumulative use of PPI was associated with an increased risk of osteoporotic fracture (p-value for trend < 0.001) and the cumulative PPI use for more than one year had a higher osteoporotic fracture risk than that of others (odds ratio (OR) = 1.42, 95% CI = 1.32 to 1.52) [16]. Also, when compared to histamine-2 receptor antagonist (H2RA) users, PPI users had a higher odds ratio (OR) of osteoporotic fracture (OR = 1.11, 95% CI = 1.08 to 1.13), and regular PPI users for one year had a higher risk of osteoporotic fracture than H2RA users (OR = 1.37, 95% CI = 1.26 to 1.50) [16].

Fusaro et al. conducted a cohort study in hemodialysis patients and reported a significantly higher incidence rate of fractures other than hip (subdistribution hazard ratio [SHR] = 1.36; 95% CI = 1.24 to 1.51; p < 0.001) and hip fractures (SHR = 1.70; 95% CI = 1.43 to 2.02; p < 0.001) in PPI-treated group when compared to the non-PPI group and suggested that around one-half of hip fractures and more than one-third of bone fractures other than hip may be prevented by eliminating PPI use in treated patients [17]. Among type 2 diabetes mellitus (T2DM) patients, a significant association between PPI use and increased hip fracture risk was noted by Chou et al. with an adjusted HR of 1.41 (95% CI = 1.29 to 1.54; p < 0.001) but did not observe a significant dose-response relationship [18]. Freedberg et al. in a case-control study observed a significant increase in fracture risk with PPI use in young adults aged 18 to 29 years (adjusted OR = 1.39, 95% CI = 1.26 to 1.53) but reported no significant association in children less than 18 years of age (adjusted OR = 1.13, 95% CI = 0.92 to 1.39) [19]. In contrast, Harding et al. performed a cohort study and observed no appreciable association between PPI use and non-vertebral fracture risk among light, moderate, and heavy PPI users with adjusted HRs of 1.08 (95% CI = 0.83 to 1.40), 1.30 (95% CI = 0.86 to 1.95), and 0.95 (95% CI = 0.68 to 1.34), respectively [26]. Thus, while *H. pylori* infection causing PUD may lead to osteoporosis, the medication used for PUD management may also play an important role in osteoporosis. Therefore, cautious use of PPI is warranted, especially in a high-risk population, to prevent the occurrence of osteoporotic fracture. Table 2 summarizes the findings of the studies mentioned above.

**Table 2:** Summary of Studies Discussing the Association Between PUD, H. pylori Infection, and PPI Use With Osteoporosis PPI: proton-pump inhibitor; T2DM: type 2 diabetes mellitus; H2RA: histamine-2 receptor antagonist; *H. pylori: Helicobacter pylori*; PUD: peptic ulcer disease; RCT: randomized controlled trial.

Study	Author	Year	Type of study	Patients	Purpose of study	Results	Conclusion
1	Chou et al. [18]	2020	Cohort	44,341	To elucidate the relation between PPI use and hip fracture risk in T2DM patients of Taiwan.	PPI group showed a significantly higher incidence and risk of hip fracture than non-PPI group in T2DM patients. But no significant dose-response relationship was found between PPI use and hip fracture.	Among T2DM patients, PPI use was significantly associated with hip fracture risk.
2	Park et al. [16]	2020	Case-control	59,240	To investigate the risk of osteoporosis with the duration and regular use of PPI, and to compare osteoporosis risk between PPI and H2RA use.	A significant association between increased osteoporotic fracture risk and PPI use was found, particularly with a regular and longer duration of PPI use of more than one year. A recent one-year PPI use had a higher osteoporotic risk than that of H2RA use.	Regular as well as extended PPI use for more than one year is significantly associated with increased osteoporotic fracture risk. This risk was higher in PPI users than in H2RA users.
3	Choi et al. [12]	2019	Cohort	50,002	To evaluate the association between PUD and osteoporosis risk in the South Korean population.	A significantly higher risk of osteoporosis was found in the PUD group when compared to the control group.	PUD poses a significant risk for the development of osteoporosis.
4	Fusaro et al. [17]	2019	Cohort	27,097	To assess the association between PPI use and bone (other than hip) and hip fracture in hospitalized hemodialysis patients.	A significantly higher incidence rate of bone (other than hip) and hip fracture was noted in the PPI-treated group when compared to the non-PPI group in hemodialysis patients.	PPI was associated with a significantly higher risk of osteoporosis in hemodialysis patients. Hip and bone (other than hip) fracture rates could be reduced by avoiding PPI use in treated patients.
5	Yoon et al. [15]	2019	Cohort	3,479	To assess the association between PUD and osteoporosis in the Korean population.	Men and women in the PUD group had a greater risk and higher incidence of developing osteoporosis in comparison to the control group.	PUD is coupled with a higher incidence and risk of developing osteoporosis in both men and women.
6	Harding et al. [25]	2018	Cohort	4,438	To determine the association between PPI use and fracture risk.	No association was found between PPI use and non-vertebral fracture risk among light, moderate, and heavy PPI users.	PPI use was not associated with increased fracture risk.
7	Wu et al. [2]	2016	Cohort	27,132	To investigate osteoporosis risk in PUD patients of the Taiwan population.	PUD patients had a significantly higher risk, incidence, and a faster development of osteoporosis than non-PUD group. Furthermore, a significant association was found between PUD patients with PPI use and osteoporosis than PUD patients with no use of PPI.	The risk of osteoporosis was significantly higher and faster in PUD patients, especially with PPI use. PUD could be an early predictor of osteoporosis.
8	Shih et al. [13]	2016	Cohort	5,447	To study the incidence of osteoporosis among those who received *H. pylori* eradication therapy and the effect of early and late *H. pylori* eradication therapy on bone in the Taiwan population.	*H. pylori *infection may be associated with an increased risk of osteoporosis in the Taiwanese population. When follow-up was less than five years, early and late eradication groups had higher risks for osteoporosis. But, beyond five years, the risk was seen only in late eradication group.	*H. pylori* infection increases the risk of osteoporosis, and early *H. pylori* eradication therapy may reduce the incidence of osteoporosis.
9	Jo et al. [14]	2015	RCT	39	To determine if PPIs (irreversible binding to H^+^K^+^-ATPase) affect H^+^-ATPase on osteoclasts when compared to revaprazan (reversible binding to H^+^K^+^-ATPase).	Significant alterations in bone parameters were noted in the elderly patients who took PPI for more than eight weeks, when compared to revaprazan.	PPIs can inhibit the vacuolar-type H^+^-ATPase on osteoclasts and, in the elderly, can significantly alter bone parameters leading to a higher risk of osteoporosis.
10	Freedberg et al. [19]	2015	Case-control study	124,799	To study if PPI use is a risk factor for fracture among children and young adults (aged four to 29).	A significant association was found between PPI use and fracture risk among young adults but not in children less than 18 years of age.	PPI use increased fracture risk in young adults (18 to 29 years of age), but not in children (less than 18 years of age).

Factors Causing Osteoporosis in PUD

Hypochlorhydric/achlorhydric conditions in PUD can result in decreased absorption of Ca, Fe, Mg, and vitamin B12 [24]. *H. pylori* infection may induce an inflammatory state and can also result in hormonal imbalance [25,28,29]. 

Malabsorption: Ca is absorbed in the small intestine in the ionized form [24]. The release of ionized Ca from insoluble Ca salts is aided by the acid-rich, low pH in the stomach. As Ca plays a crucial role in bone development and bone mineral accumulation, chronic use of gastric acid suppression drugs like PPI can lead to hypochlorhydria/achlorhydria resulting in decreased enteral absorption of Ca, contributing to osteoporosis with reduced BMD [24,30,31]. However, an RCT conducted by Jo et al. noticed that both urine deoxypyridinoline (DPD) (a bone resorption marker) and serum Ca were increased after PPI use, which portrays that PPI use directly affects bone metabolism through osteoclast action rather than by decreased intestinal Ca absorption [14]. Hence, more studies are required to ascertain the relation between PPI use and Ca absorption.

Mg levels were found to be low in patients using long-term PPI. A prospective cohort study conducted by Kieboom et al. in 2015 revealed an increased risk of hypomagnesemia with PPI use (n = 36; OR = 2.00, 95% CI = 1.36 to 2.93) with serum Mg level 0.022 mEq/L lower in PPI users (n = 724; 95% CI = -0.032 to -0.014 mEq/L) versus those with no PPI use [32]. The elevated risk with PPI use was only evident after prolonged use (range = 182 to 2,618 days; OR = 2.99, 95% CI = 1.73 to 5.15). A similar association was found with H2RA users (n = 250) revealing an increased risk of hypomagnesemia (n = 12; OR = 2.00, 95% CI = 1.08 to 3.72) compared to non-users [32]. A study in hemodialysis patients conducted by Sakaguchi et al. found a significantly (p < 0.001) increased risk of hip fracture in patients with lower serum Mg levels [33]. A one milligram/deciliter (mg/dL) increase in serum Mg level led to a 14.3% decrease (95% CI = 3.8 to 23.8; p < 0.01) in incident hip fracture risk. An increase in serum Mg level up to 4.0 mg/dL was associated with a linear decrease in fracture risk [33]. The US FDA raised a similar concern in 2011 and issued a drug safety announcement regarding the potential risk of hypomagnesemia with prolonged PPI use (over one year) [34]. The relationship between hypomagnesemia and PPI use can be explained by the effect of PPI on the proper functioning of colonic transient receptor potential melastatin-6 transporters (TRPMs). TRPM cation channels 6 and 7 regulate the active transport of Mg in the gastrointestinal tract. A more acidic pH maintained by the colonic H^+^K^+^-ATPase increases the activity of these channels. The inhibition of colonic H^+^K^+^-ATPase by PPIs leads to decreased activity of TRPM cation channels, resulting in hypomagnesemia in some patients [35,36]. Mg deficiency may have direct effects on the bone, such as decreased bone stiffness, decreased osteoblast activity, and increased osteoclast activity. The indirect effects of Mg deficiency include decreased parathyroid hormone (PTH) secretion leading to vitamin D deficiency resulting in decreased bone formation. Hypomagnesemia can also promote inflammation, which can lead to increased bone resorption [37]. Table 3 summarizes the studies discussing the effect of PPI use on serum Mg levels and fracture risk.

**Table 3:** Summary of Studies Discussing the Relation Between PPI Use, Serum Mg Levels, and Fracture Risk Mg: magnesium; PPI: proton-pump inhibitor.

Study	Author	Year	Type of study	Patients	Purpose of study	Results	Conclusion
1	Sakaguchi et al. [33]	2018	Cohort	113,683	To assess the association of serum Mg levels and risk of incident hip fracture in hemodialysis patients.	The crude hip fracture incidence rate was significantly higher in patients with lower serum Mg levels for both men and women. There was a linear decrease in fracture risk with increasing serum Mg levels.	Hypomagnesemia is associated with a significantly increased risk of fracture.
2	Kieboom et al. [32]	2015	Cohort	9,818	To analyze the relation between PPI use and hypomagnesemia risk.	Serum Mg level was lower in PPI users versus non-users. PPI use was associated with a higher risk of hypomagnesemia when compared to no use.	PPI use for more than six months was associated with lower serum Mg levels versus with no use.

Vitamin B12 absorption essentially requires the acidic pH of the stomach [38]. Therefore, gastric acid inhibition by PPI may inhibit the absorption of vitamin B12 and can result in low levels of vitamin B12 [39,40]. Vitamin B12 deficiency can lead to an increase in osteoclastic activity, a decrease in osteoblastic activity, and homocysteinemia causing abnormal collagen cross-linking [11]. The deficiency of vitamin B12 can affect bone fragility through modulation of collagen cross-linking independently of areal BMD [41,42]. This increased bone fragility, along with increased fall risk in elderly patients with PPI use due to peripheral neuropathy caused by vitamin B12 deficiency, can contribute to increased fracture rates [42,43].

Inflammation: In PUD, *H. pylori* infection elicits a localized and systemic inflammatory response that may cause a rise in levels of several cytokines, including TNF-α, IL-1, and IL-6 [28]. Chronic inflammatory processes promote osteoclastogenesis as cytokines enhance osteoclast formation and development, stimulating bone resorption [44].

Endocrine: Regarding hormonal factors, chronic hypergastrinemia can occur as a result of hypochlorhydria/achlorhydria caused by *H. pylori* infection and chronic use of potent gastric antisecretory drugs like PPI [23,25]. Hypergastrinemia induced by PPI use can lead to parathyroid hyperplasia, resulting in increased PTH secretion and decreased BMD [24,45].

A decrease in total, free, and bioavailable estradiol levels was found in patients of both genders with *H. pylori* infection by CagA-positive strain in a study conducted by Gennari et al. [29]. The gastric parietal cells express aromatase enzyme and aid in the peripheral conversion of androgen to estrogen. The loss of gastric parietal cells due to *H. pylori* infection can thus lead to a decrease in the pool of estrogen, causing a potential link to bone fragility [29,46].

Ghrelin levels were significantly (p < 0.001) lower in CagA-positive patients than in CagA-negative and *H. pylori-*uninfected patients, both in the fasting state and after the meal, in a cohort study by Gennari et al. [29]. *H. pylori* infection can destroy gastric oxyntic glands that secrete ghrelin. Ghrelin, besides appetite stimulation, promotes osteoblast proliferation and differentiation. Lower ghrelin levels can decrease bone formation and increase the risk of bone fracture [29,47]. Table 4 summarizes the study findings between *H. pylori* infection with CagA-positive strain and the effect on estrogen and ghrelin levels.

**Table 4:** Summary of the Study Outlining the Association Between CagA-Positive H. pylori Infection and Decreased Levels of Ghrelin and Estrogen Levels *H. pylori: Helicobacter pylori*.

Study	Author	Year	Type of study	Patients	Purpose of study	Results	Conclusion
1	Gennari et al. [29]	2021	Cohort	1,149	To evaluate the influence of CagA-positive *H. pylori* infection on bone fragility.	*H. pylori* infection with CagA-positive strain was associated with a higher risk of fracture. The prominent reasons behind this association included an increase in proinflammatory cytokines, reduction in total, free, and bioavailable estradiol, reduced ghrelin level, and increased level of postprandial serotonin.	*H. pylori* infection by CagA-positive strain can lead to osteoporosis and increased rate of incident fractures. Decreased level of circulating estradiol and ghrelin along with an increase in postprandial serotonin level and enhanced inflammatory state contributes to osteoporosis.

**Figure 2:**
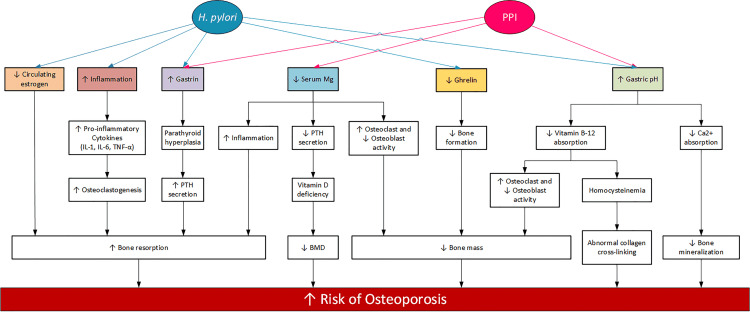
Mechanisms by Which Osteoporosis Is Caused by H. pylori Infection and PPI Use *H. pylori: Helicobacter pylori*; PPI: proton-pump inhibitor; IL-1: interleukin-1; IL-6: interleukin-6; TNF-α: tumor necrosis factor alpha; PTH: parathyroid hormone; Ca: calcium; BMD: bone mineral density.

The above studies and findings portray the primary mechanisms by which osteoporosis emerges as a complication of PUD, *H. pylori* infection, and PPI use. PUD and PPI use may lead to the release of proinflammatory cytokines and malabsorption of nutrients like Ca, Mg, and vitamin B12. It can also cause hormonal imbalance, including increased gastrin secretion and reduced ghrelin and circulating estrogen levels. These may lead to the development of osteoporosis. Figure 2 briefly outlines the mechanisms by which *H. pylori* infection and PPI use can result in an increased risk of osteoporosis and fracture.

Limitations

There were limitations to our study. Most of the studies included in this systematic review were observational studies and contained only one RCT. Therefore, the risk of potential bias is not negligible. Also, we identified articles that were published only in the English language in this systematic review.

## Conclusions

In this systematic review, we tried to elucidate the link between PUD and fracture risk. From the available data, we conclude that PUD, *H. pylori* infection (particularly, the more virulent CagA-positive strain), and PPI use may result in osteoporosis through different mechanisms, including malabsorption of nutrients, increase in proinflammatory cytokines, and creating an endocrinal imbalance. We observed a lower incidence of osteoporosis with early *H. pylori* eradication therapy. Therefore, early management of *H. pylori *infection should be implemented for patients with a high risk for osteoporosis. Also, we noted that long-term regular use of PPI for over one year had a more adverse effect on BMD. Hence, physicians should be cautious while prescribing long-term PPIs for patients, particularly the elderly population who are at elevated risk of fracture. However, further RCTs should be conducted to establish a causal relationship between PUD and osteoporosis and to understand the underlying mechanisms.
